# Supplementary material for: Trends and age-period-cohort analysis of migraine incidence in China from 1990 to 2021
Source: PLoS One. 2026 Feb 13;21(2):e0338930. doi: 10.1371/journal.pone.0338930 (PMC12904424; doi:10.1371/journal.pone.0338930)
Supplement: S1 File — This file provides the step-by-step description of the decomposition approach used to attribute changes in migraine incidence, prevalence, and YLDs to population growth, population ageing, and epidemiological change. (DOCX)s [file pone.0338930.s001.docx]

Supplementary Text S1. The Decomposition Method

Conceptual framework

We followed a robust decomposition analysis method described in previous studies (e.g., Cheng et al. 2020; Zhu et al. 2024), which has been widely applied in global burden analyses. This approach attributes the overall change in cases or rates between two time points to three components: population growth, population ageing, and epidemiological change. It is robust to the choice of decomposition order and reference year, ensuring reproducibility and comparability across studies.

Notation and definitions

Let the population be divided into I age groups (and stratified by sex when appropriate). For age group i at year j (j=1,2):

dij: number of migraine cases (or YLDs)
n­ij­: population size
mij = $\frac{d_{ij}}{n_{ij}}$age-specific rate (incidence, prevalence, or YLD rate)
sij =$\frac{n_{ij}}{N_{j}}$: age structure (proportion of population)
Nj =$\sum_{i} n_{ij}$: total population size
Dj =$\sum_{i} d_{ij}$: total number of cases
Mj =$\frac{d_{j}}{n_{j}}$: crude rate

The total change in cases (or burden) between baseline (t1) and comparison year (t2) is:

ΔD = D2 - D1

Main effects

$$M_{P}=\sum_{i=1}^{I} (N2-N1)s_{i1}m_{i1}$$

$$M_{a}=\sum_{i=1}^{I} N1(s_{i2}-s_{i1})m_{i1}$$

$$M_{m}=\sum_{i=1}^{I} N_{1}s_{i1}(m_{i2}-m_{i1})$$

where Mp represents the effect of population growth, Ma the effect of population ageing, and Mm the effect of epidemiological change.

Interaction terms

$$I_{pa}=\sum_{i=1}^{I} (N_{2}-N_{1})(s_{i2}-s_{i1})m_{i1}$$

$$I_{pm}=\sum_{i=1}^{I} \left( N_{2}-N_{1} \right)s_{i1}(m_{i2}-m_{i1})$$

$$I_{am}=\sum_{i=1}^{I} N_{1}(s_{i2}-s_{i1})(m_{i2}-m_{i1})$$

$$I_{pam}=\sum_{i=1}^{I} \left( N_{2}-N_{1} \right)(s_{i2}-s_{i1})(m_{i2}-m_{i1})$$

Final allocation

To avoid dependence on the order of decomposition, the interactions were equally distributed among the three components:

P = M_p_ + $\frac{1}{2}$ Ipa + $\frac{1}{2}$ Ipm + $\frac{1}{3}$ Ipam
A = M_a_ +$\frac{1}{2}$ Ipa + $\frac{1}{2}$ Iam + $\frac{1}{3}$ Ipam
M = M_m_ +$\frac{1}{2}$ Ipm + $\frac{1}{2}$ Iam + $\frac{1}{3}$ Ipam

Thus,

ΔD = P + A + M,

where P is the contribution of population growth, A is the contribution of ageing, and M is the contribution of epidemiological change.

Application in this study

This framework was applied separately to incidence, prevalence, and YLDs of migraine in China from 1990 to 2021, stratified by sex. Both absolute contributions (numbers of cases or changes in YLDs) and relative contributions (percentage of total change) were calculated. All calculations were conducted in R software (version 4.4.1).
