# Supplementary material for: Trends and age-period-cohort analysis of migraine incidence in China from 1990 to 2021
Source: PLoS One. 2026 Feb 13;21(2):e0338930. doi: 10.1371/journal.pone.0338930 (PMC12904424; doi:10.1371/journal.pone.0338930)
Supplement: S2 File — This file contains the dataset used for calculating the incidence, prevalence, and YLDs of migraine in China, as well as the corresponding inputs for the decomposition and age-period-cohort analyses. (ZIP) [file pone.0338930.s002.zip › raw data/file declaration.docx]

The data used in this study were obtained from the Global Burden of Disease (GBD) database maintained by the Institute for Health Metrics and Evaluation (IHME). These data are publicly available, aggregated estimates and do not contain individual-level human participant information.

The original GBD data were processed using R software to extract China-specific estimates and to conduct the statistical analyses required for this study. These intermediate processed datasets were used solely for internal data management and analysis and are not shared as Supporting Information.

The Supporting Information files provided with this manuscript include only fully anonymized, aggregated result data that underlie the tables and figures presented in the manuscript, as detailed below:

Margine_china_number and Margine_china_Rate: Aggregated data corresponding to all values reported in Table 1, derived from the publicly available GBD estimates using R software.

Joinpoint folder: Aggregated data underlying Table 2, generated using R software and Joinpoint regression analysis based on GBD-derived estimates.

YLDs (Years Lived with Disability) Decomposition analysis: Aggregated data supporting Figure 2, obtained through decomposition analysis of GBD-derived estimates using R software.

All Supporting Information files contain only summary-level data and do not include any personally identifiable or sensitive information.
