# Supplementary figures and images for: Trends and age-period-cohort analysis of migraine incidence in China from 1990 to 2021
Source: PLoS One. 2026 Feb 13;21(2):e0338930. doi: 10.1371/journal.pone.0338930 (PMC12904424; doi:10.1371/journal.pone.0338930)

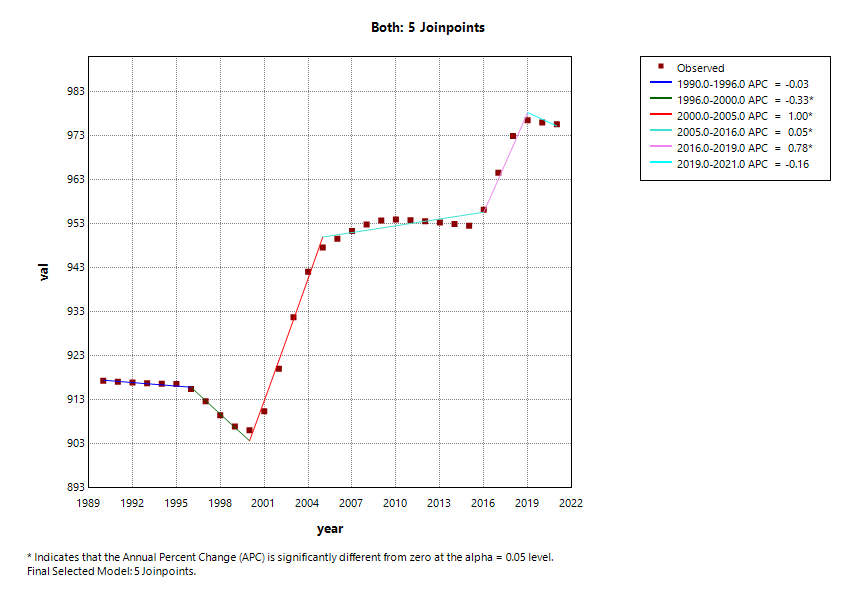

Supplement: S2 File — This file contains the dataset used for calculating the incidence, prevalence, and YLDs of migraine in China, as well as the corresponding inputs for the decomposition and age-period-cohort analyses. (ZIP) [file pone.0338930.s002.zip › raw data/joinpoint/joinpoint/Incidence/export both.Export.Graph.tiff]

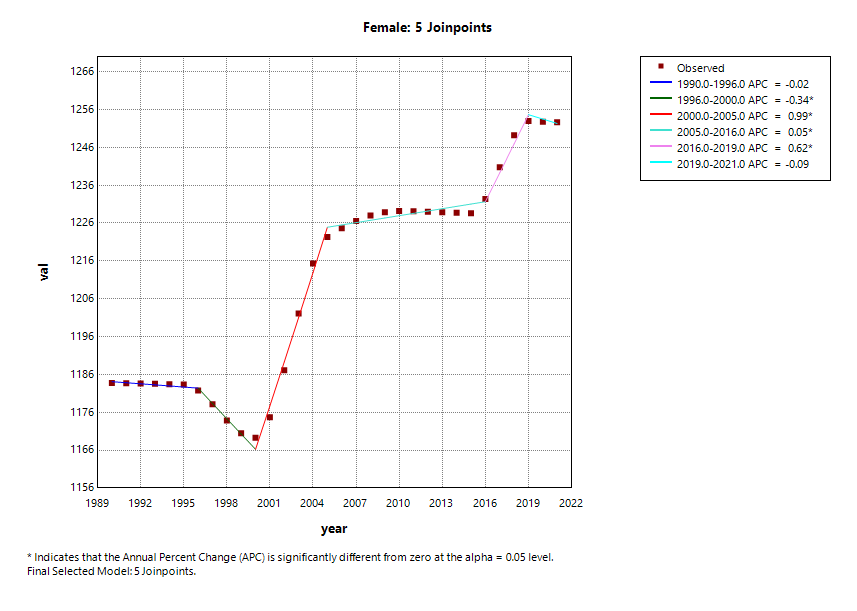

Supplement: S2 File — This file contains the dataset used for calculating the incidence, prevalence, and YLDs of migraine in China, as well as the corresponding inputs for the decomposition and age-period-cohort analyses. (ZIP) [file pone.0338930.s002.zip › raw data/joinpoint/joinpoint/Incidence/export female.Export.Graph.tiff]

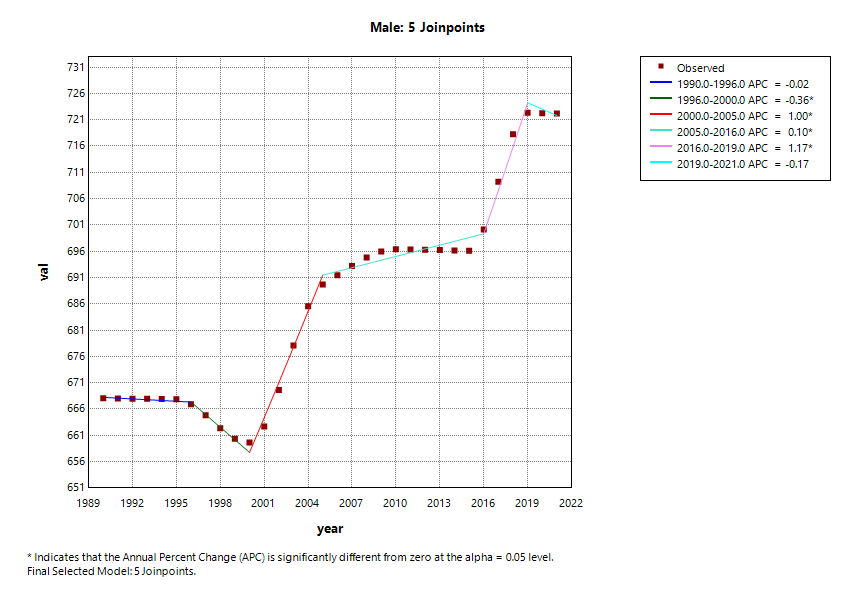

Supplement: S2 File — This file contains the dataset used for calculating the incidence, prevalence, and YLDs of migraine in China, as well as the corresponding inputs for the decomposition and age-period-cohort analyses. (ZIP) [file pone.0338930.s002.zip › raw data/joinpoint/joinpoint/Incidence/export male.Export.Graph.bmp]

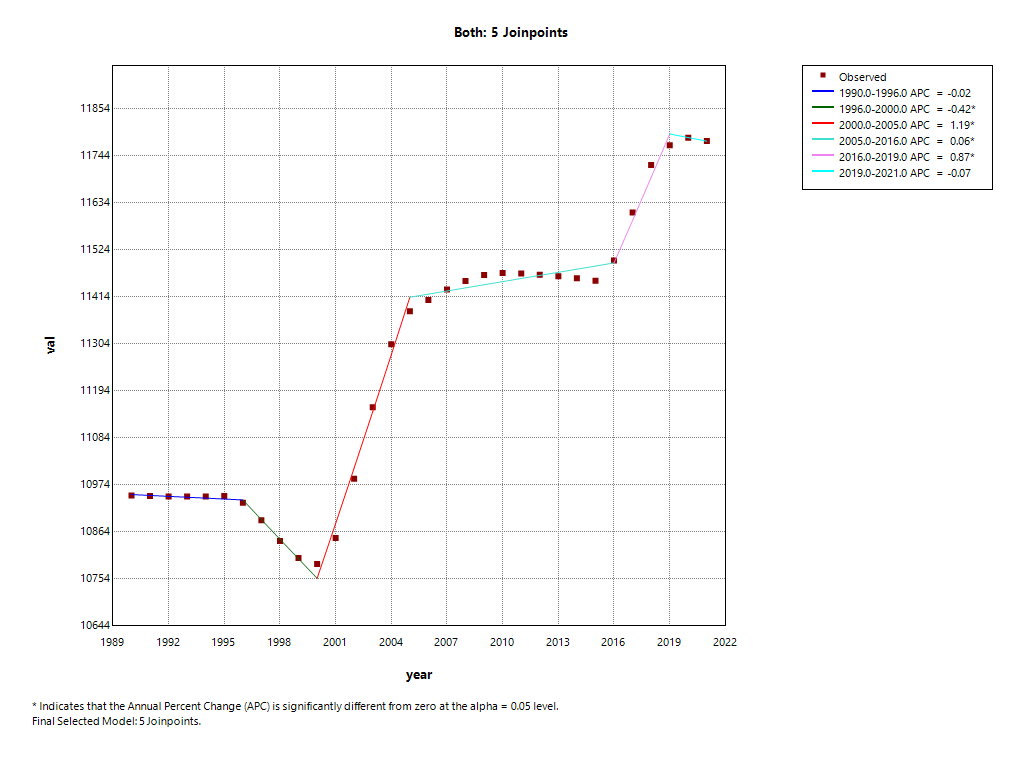

Supplement: S2 File — This file contains the dataset used for calculating the incidence, prevalence, and YLDs of migraine in China, as well as the corresponding inputs for the decomposition and age-period-cohort analyses. (ZIP) [file pone.0338930.s002.zip › raw data/joinpoint/joinpoint/Prevalence/export both.Export.Graph.tiff]

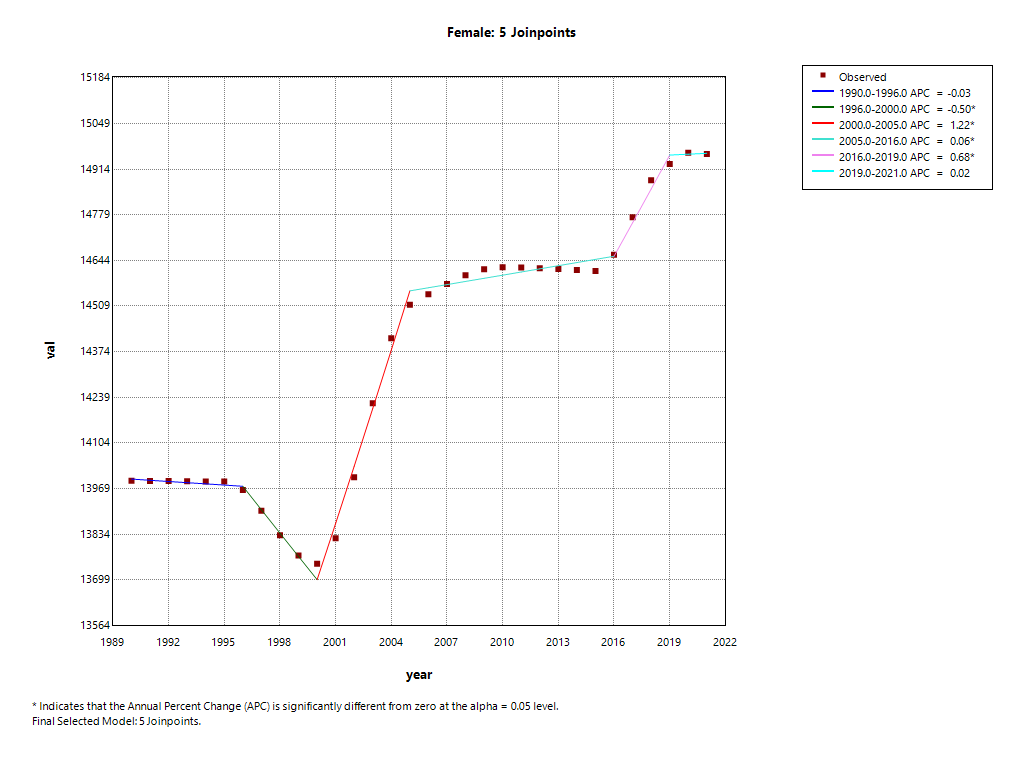

Supplement: S2 File — This file contains the dataset used for calculating the incidence, prevalence, and YLDs of migraine in China, as well as the corresponding inputs for the decomposition and age-period-cohort analyses. (ZIP) [file pone.0338930.s002.zip › raw data/joinpoint/joinpoint/Prevalence/export female.Export.Graph.tiff]

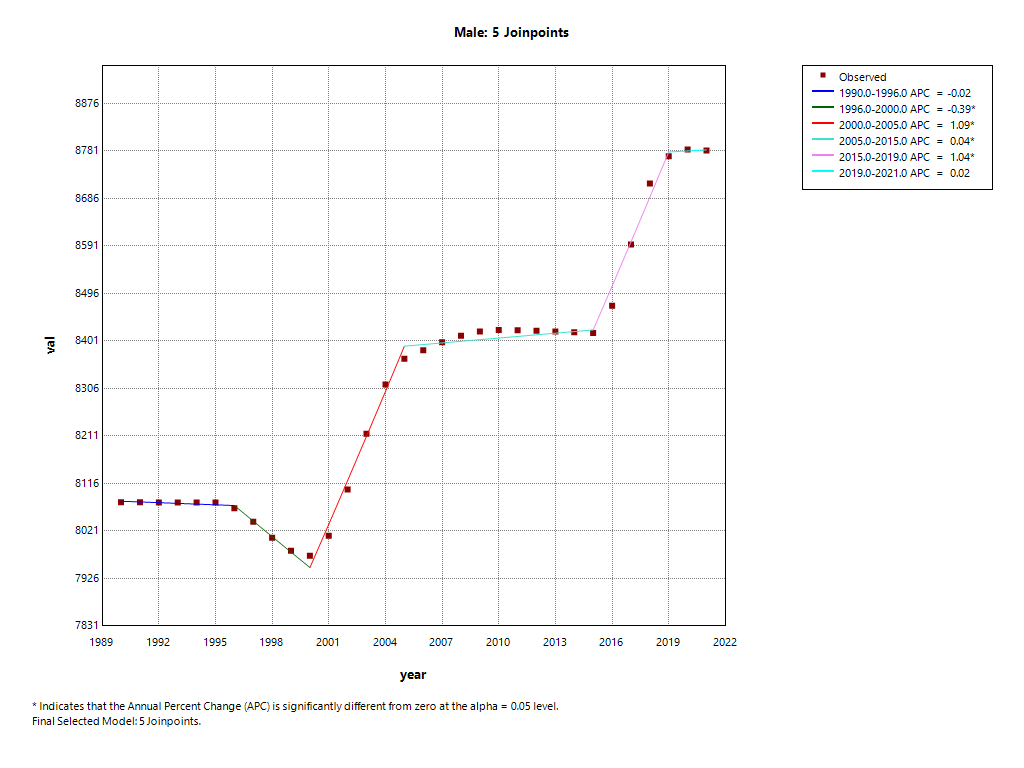

Supplement: S2 File — This file contains the dataset used for calculating the incidence, prevalence, and YLDs of migraine in China, as well as the corresponding inputs for the decomposition and age-period-cohort analyses. (ZIP) [file pone.0338930.s002.zip › raw data/joinpoint/joinpoint/Prevalence/export male.Export.Graph.tiff]

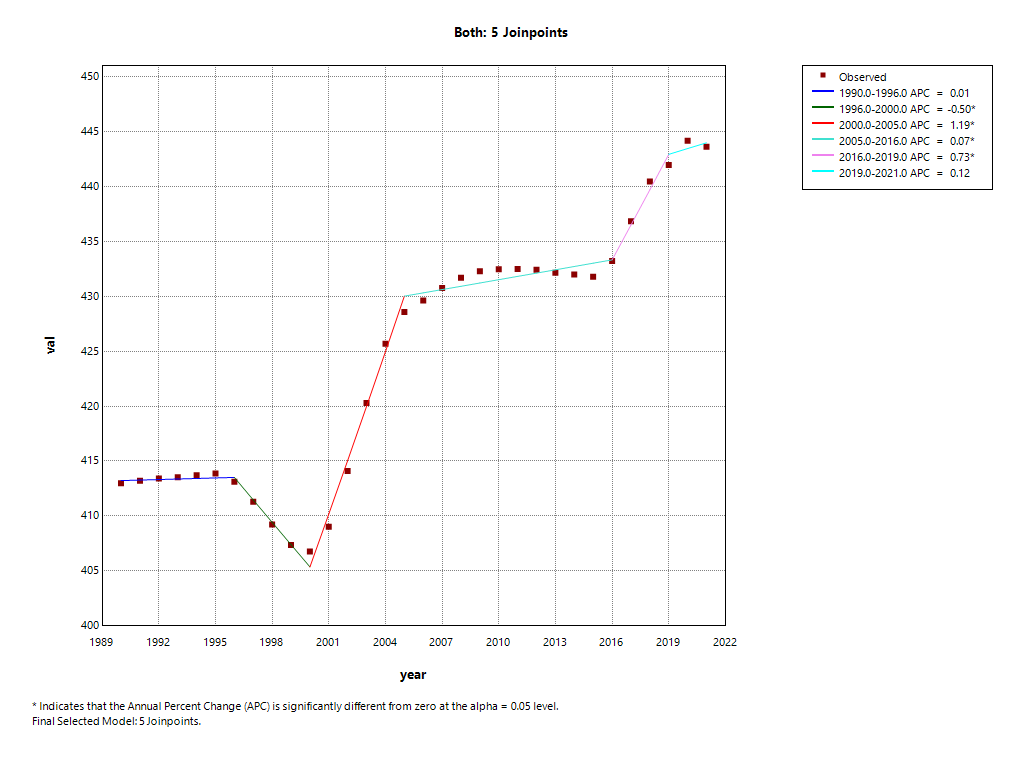

Supplement: S2 File — This file contains the dataset used for calculating the incidence, prevalence, and YLDs of migraine in China, as well as the corresponding inputs for the decomposition and age-period-cohort analyses. (ZIP) [file pone.0338930.s002.zip › raw data/joinpoint/joinpoint/YLDs/export both.Export.Graph.tiff]

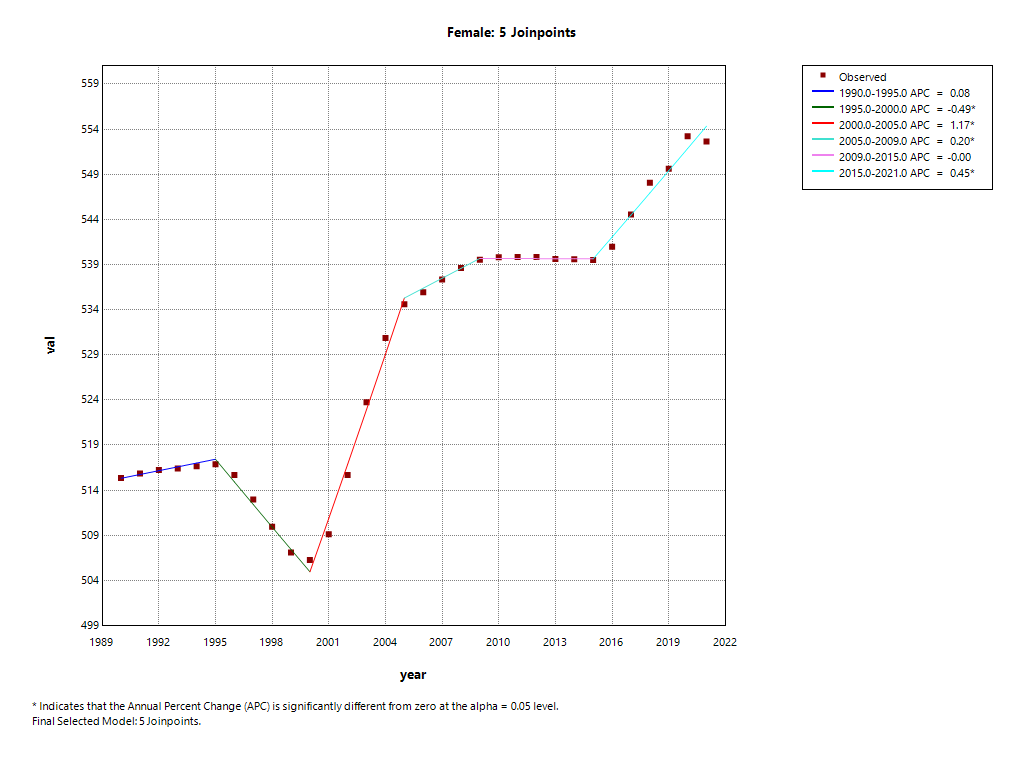

Supplement: S2 File — This file contains the dataset used for calculating the incidence, prevalence, and YLDs of migraine in China, as well as the corresponding inputs for the decomposition and age-period-cohort analyses. (ZIP) [file pone.0338930.s002.zip › raw data/joinpoint/joinpoint/YLDs/export female.Export.Graph.tiff]

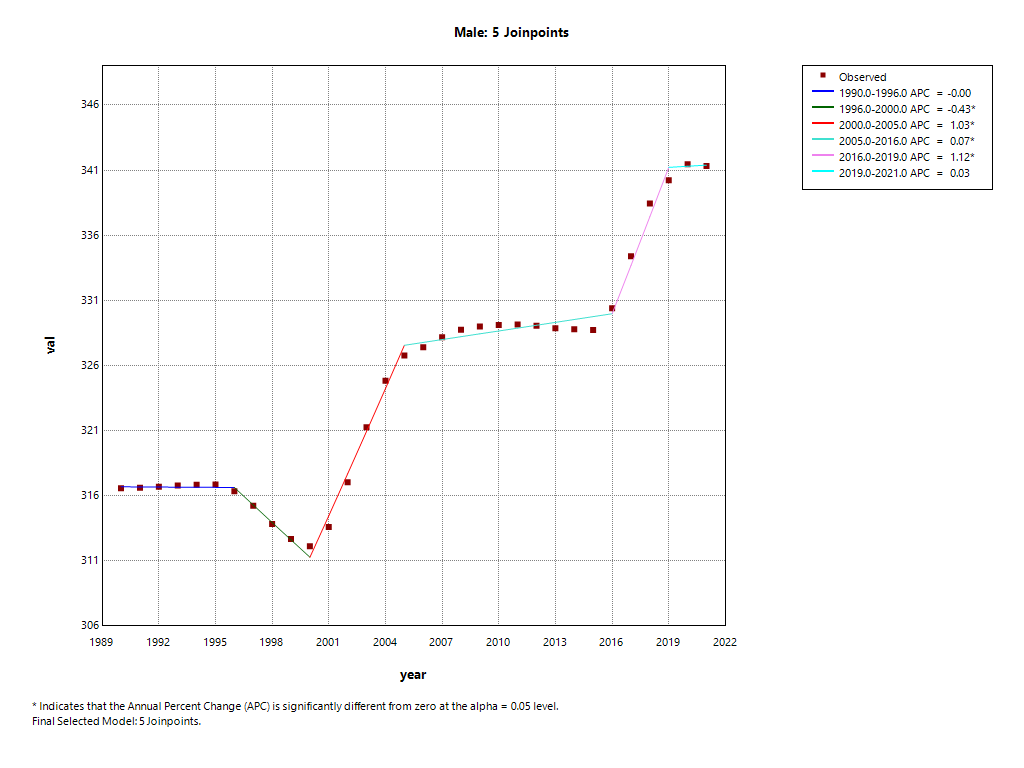

Supplement: S2 File — This file contains the dataset used for calculating the incidence, prevalence, and YLDs of migraine in China, as well as the corresponding inputs for the decomposition and age-period-cohort analyses. (ZIP) [file pone.0338930.s002.zip › raw data/joinpoint/joinpoint/YLDs/export male.Export.Graph.tiff]
