# Supplementary material for: Trends and age-period-cohort analysis of migraine incidence in China from 1990 to 2021
Source: PLoS One. 2026 Feb 13;21(2):e0338930. doi: 10.1371/journal.pone.0338930 (PMC12904424; doi:10.1371/journal.pone.0338930)
Supplement: Attachment — Submitted filename: Author_Response_Letter.docx [file pone.0338930.s004.docx]

Manuscript Number : PONE-D-25-16628

Title: Trends and age-period-cohort analysis of migraine incidence in China from 1990 to 2021

Thank you for giving us the opportunity of reviewing our manuscript. A revised version of the manuscript has been generated where all reviewers' comments have been taken into account. Those comments are all valuable and very helpful for revising and improving our paper. Our replies to the reviewers comments are reported hereafter. All changes with respect to the previous submission have been highlighted in the PDF file of the manuscript.

**Reviewer#1 point 1: A.** Introduction Needs Strengthening

The introduction lacks critical context. It should:

Specify gaps in prior Chinese or GBD migraine trend analyses,

Clarify the motivation for focusing on incidence,

Briefly acknowledge limitations inherent in using modeled data.

Recommendation: Expand the introduction to articulate the scientific rationale and relevance of the research more clearly.

Author response:

We thank the reviewer for this insightful suggestion. We have substantially revised the Introduction to strengthen the scientific rationale and relevance of our study.

1. Original text (before revision):

“However, there is a lack of long-term trend studies specifically targeting different populations in China, particularly a systematic analysis of age, period, and cohort effects.”

(Original manuscript, Lines 60–61)

This version did not clearly specify the research gaps, the motivation for focusing on incidence, or the limitations of using modeled data.

2. Revised text (after revision):

“The incidence of migraine, that is, the number of new cases, can more accurately reflect the change of risk over time than the total number of patients. Although this is very important for making preventive measures and policy planning, the continuous monitoring of migraine incidence in China is still insufficient. Although many studies around the world have discussed the burden of migraine, few studies can make a detailed analysis according to the characteristics of these people, such as age, gender or birth year[7]. Moreover, the scope and methods of our existing Chinese research are very narrow, which makes it difficult for us to accurately judge the long-term development trend[9-11]. Many studies conflate incidence, prevalence, and YLDs, potentially undermining epidemiological clarity.”

(Revised manuscript, Lines 62–71)

“While modeled estimates enable extensive temporal analyses, discrepancies from observed diagnoses may arise due to heterogeneity in input sources and modeling parameters..”

(Revised manuscript, Lines 89–90)

“First, all estimates were generated from the Global Burden of Disease (GBD) database, which, although incorporating Chinese data sources—surveys, registries, hospital records, and insurance claims—relies on statistical modeling rather than direct data analysis. As a result, the outcomes may not precisely represent primary national data, and biases arising from input quality or model specification could yield systematic error.”

(Revised manuscript, Lines 306–311)

“Finally, although the age-period-cohort model we use can help us see the law of time change, some settings of this model itself make it impossible for us to directly judge the causal relationship between things”

(Revised manuscript, Lines 318–320)

3. Explanation of changes:

Research gap specified: We added that previous GBD and Chinese studies lack stratified, long-term trend analyses (Lines 62–71).

Motivation for focusing on incidence: We explicitly stated that incidence better reflects new risk patterns than prevalence and is critical for prevention and policy planning (Lines 62–64).

Limitations acknowledged: We briefly noted that GBD relies on modeled estimates, which may differ from observed diagnoses (Lines 89-90; Lines 306–311;Lines 318–320).

4. Purpose and significance:

These revisions provide a clearer rationale for the study, emphasizing both its novelty and its relevance to public health. By clarifying research gaps, justifying the focus on incidence, and transparently acknowledging data limitations, we believe the revised Introduction now aligns more closely with the reviewer’s recommendation and improves the manuscript’s clarity and rigor.

**Reviewer #1 point 2: B**. Terminological Confusion: Incidence, Prevalence, and YLDs

The manuscript frequently conflates incidence, prevalence, and YLDs. This undermines epidemiological clarity.

Recommendation: Clearly define each metric and apply terminology consistently.

Author response:

We thank the reviewer for pointing out this critical issue. We have carefully revised the manuscript to clearly define and consistently apply the terms incidence, prevalence, and YLDs throughout the text.

1. Original text (before revision):

“Joinpoint regression analysis was used to evaluate the temporal trend of migraine prevalence, incidence, and years lived with disability (YLDs), and the age–period–cohort (APC) model was used to evaluate the effects of age, time and birth cohort.”

(Original manuscript, Lines 31–33)

In the original text, incidence, prevalence, and YLDs were often mentioned together without distinction, and their epidemiological meanings were not clearly defined.

2. Revised text (after revision):

“Incidence: The occurrence of new migraine cases and captures temporal shifts in risk more directly than prevalence. Prevalence: The total number of existing cases at a specific time, reflecting the population burden. YLDs: Years lived with disability, measure the health loss due to migraine-related disability, combining prevalence with disability weights. In this study, we examined these three indicators separately to ensure clarity.”

(Revised manuscript, Lines 144–146)

“APC: Annual Percentage Change; AAPC: Average Annual Percentage Change；ASIR：Age-Standardized Incidence Rate; ASPR: Age-Standardized Prevalence Rate; ASYR: Age-Standardized YLDs Rate”

(Revised manuscript, Lines154-156)

3. Explanation of changes:

Definitions clarified: We added explicit definitions of incidence, prevalence, and YLDs in the notes of Table 1 (Lines 144–146).

Consistent application: We revised all tables, figures, and results to report ASIR, ASPR, and ASYR separately (Lines 154-156).

4. Purpose and significance:

These revisions ensure epidemiological clarity and address the reviewer’s concern. By defining each metric explicitly and using them consistently throughout, the manuscript now avoids confusion and enhances interpretability for both researchers and public health practitioners.

**Reviewer #1 point 3: C.** Critical Omission: Definition of Migraine in GBD

The manuscript does not describe how migraine cases were defined in the GBD 2021 model. As migraine diagnosis is clinical and symptom-based (e.g., per ICHD-3), clarity is essential.

Recommendation: Specify:

Whether ICHD-2 or ICHD-3 was used,

The types of source data for China (e.g., surveys, claims),

How modeling handled case ascertainment and uncertainty,

The implications of using modeled incidence vs. observed diagnosis.

Author response:

We thank the reviewer for highlighting this critical omission. In response, we have revised the Methods: Data Sources section to clearly describe how migraine was defined in GBD 2021, the data sources used for China, the modeling framework, and its implications.

1. Original text (before revision):

“This study is based on the GBD2021 data, which provides the most recent epidemiological estimates on disease and injury burden for 21 GBD regions and 204 countries and territories from 1990 to 2021. All data can be accessed via the Global Health Data Exchange (https://vizhub.healthdata.org/gbd-results/). The dataset used in this study covers disease burden indicators, such as prevalence, incidence, and YLDs, related to migraine for different gender groups in China from 1990 to 2021.”

(Original manuscript, Lines 70–75)

This version lacked details on diagnostic criteria, Chinese data sources, and the modeling approach.

2. Revised text (after revision):

“Our research uses the data of GBD 2021. GBD 2021 provides the latest estimates of diseases and injuries in 21 disease-burden regions and 204 countries and regions from 1990 to 2021. All the data can be found publicly on the website of Global Health Data Exchange (https://vizhub.healthdata.org/gbd-results/). In GBD 2021, migraine was defined in accordance with the diagnostic criteria outlined in the International Classification of Headache Disorders, third edition (ICHD-3)[12] , without differentiating between migraine with and without aura. For us in China, the input data comes from many places, including door-to-door population surveys, registration offices dedicated to recording certain diseases, and medical system databases, such as hospital records and medical insurance reimbursement lists[6, 13]. The estimates were produced using the DisMod-MR 2.1 Bayesian meta-regression framework, which integrates heterogeneous data sources, enforces standardized case definitions, adjusts for recognized biases, and quantifies uncertainty through 95% uncertainty intervals[6]. This study utilized data on migraine-related indicators—prevalence, incidence, and YLDs—disaggregated by sex for China between 1990 and 2021. While modeled estimates enable extensive temporal analyses, discrepancies from observed diagnoses may arise due to heterogeneity in input sources and modeling parameters..”

(Revised manuscript, Lines 76–90)

3. Explanation of changes:

Diagnostic criteria: Added explicit clarification that GBD 2021 used ICHD-3 criteria (Line 79-81).

Data sources for China: Specified that Chinese input data came from household surveys, disease-specific registries, hospital records, and insurance claims (Lines 81–84).

Modeling framework: Added description of the DisMod-MR 2.1 Bayesian meta-regression model, including case definition standardization, bias correction, and uncertainty quantification (Lines 84–87).

Limitations: Noted that modeled incidence may differ from observed diagnoses due to data heterogeneity and assumptions (Lines 89–90).

4. Purpose and significance:

These revisions provide transparency on how migraine was defined and modeled in GBD 2021, directly addressing the concerns. By specifying diagnostic criteria, data sources, modeling methods, and limitations, the manuscript now offers greater methodological clarity and allows readers to better interpret the results in context.

**Reviewer #1 point 4: D.** Insufficient Methodological Transparency in APC Modeling

There is no mention of how the APC identifiability problem was handled, nor are any diagnostics reported.

Recommendation: Provide:

Identification strategy (e.g., Holford, constraint-based),

Software used,

Model diagnostics (e.g., residual analysis, AIC/BIC, VIF).

Author response:

We thank the reviewer for this valuable comment. In the revised manuscript, we have expanded the Methods: Statistical Analysis section to clarify the APC modeling strategy, the software platform used, and the limitations regarding diagnostics.

1. Original text (before revision):

“R software version 4.4.1 was employed for data visualization, decomposition analysis, and age–period–cohort (APC) analysis. Joinpoint software was used to compute the annual percent change (APC), average annual percent change (AAPC), and 95% confidence intervals (CI) for the migraine disease burden.”

(Original manuscript, Lines 77–80)

This version only listed the software and did not describe the APC identifiability strategy or diagnostics.

2. Revised text (after revision):

“Age–Period–Cohort (APC) analysis was performed using the Biowinford online platform (http://biowinford.site:3838/trial/). The platform’s identification strategy employs the constraint-based Holford method, which resolves the inherent linear dependency among age, period, and cohort effects by imposing parameter constraints on period and cohort terms. This approach aligns with the methodology implemented in the NIH’s APC web tool (https://analysistools.cancer.gov/apc/), though the NIH version is broader in scope and not tailored to migraine or GBD-specific data. The Biowinford output excludes certain model diagnostics, including residual analysis, Akaike Information Criterion (AIC), Bayesian Information Criterion (BIC), and Variance Inflation Factor (VIF). Although this represents a methodological limitation, the consistency of the identification strategy, case definitions, and data sources with established APC frameworks supports the robustness and interpretive value of the resulting trend estimates.”

(Revised manuscript, Lines 117–127)

3. Explanation of changes:

Identification strategy: We specified that the constraint-based Holford method was used to resolve the identifiability problem （Lines 118–120).

Software platform: We clarified that APC analysis was performed using the Biowinford platform (http://biowinford.site:3838/trial/), consistent with NIH’s APC web tool (Lines 120–123).

Diagnostics: We acknowledged that diagnostics such as residual analysis, AIC/BIC, and VIF were not available on this platform (Lines 123–125).

4. Additional Author Note (regarding diagnostics):

We acknowledge the reviewer’s concern regarding the absence of diagnostic outputs. The Biowinford platform, like the NIH APC tool, does not generate residuals, AIC/BIC, or VIF values. This is a limitation of the platform rather than an omission in our study. Importantly, the constraint‐based Holford method has been widely validated and applied in epidemiological studies, including those conducted under the GBD framework. Therefore, despite the lack of formal diagnostic statistics, the APC results are considered robust, reproducible, and aligned with internationally accepted practices.

5. Purpose and significance:

These revisions substantially improve methodological transparency. By specifying the identification strategy, platform, and limitations, we ensure that readers and reviewers can better understand and assess the reliability of our APC modeling results.

**Reviewer #1 point 5: E.** Joinpoint Analysis Lacks Historical or Clinical Context

Timepoint changes (e.g., in 2000, 2005, 2016) are presented without connecting them to relevant clinical or policy developments in China.

Recommendation: Contextualize Joinpoint inflection years using historical changes in health policy, treatment availability (e.g., triptans, CGRP), or diagnostic practices.

Author response:

We appreciate the reviewer’s valuable suggestion. In the revised manuscript, we have expanded the Discussion section to interpret the Joinpoint regression results in the context of relevant diagnostic practices, treatment availability, and health policy developments in China.

1. Original text (before revision):

“Based on the Joinpoint regression analysis of the age-standardized incidence rate (ASIR), age-standardized prevalence rate (ASPR), and age-standardized YLDs rate (ASYR) for migraine in China from 1990 to 2021, a downward trend was observed from 1996 to 2000, followed by an increase between 2000 and 2005, a slower growth from 2005 to 2016, acceleration from 2016 to 2019, and a slight decline from 2019 to 2021.”

(Original manuscript, Lines 108–115)

This version listed numerical trends but did not link inflection points to historical or clinical context.

2. Revised text (after revision):

“In order to find out the temporal trend of migraine burden, we analyzed the long-term data in different historical periods. From the mid-1990s to the early 2000s, the incidence and prevalence of migraine decreased slightly, which may be due to the problems of insufficient diagnosis and frequent misdiagnosis in primary health care, where the diagnostic accuracy was only 13%-18%, and people seldom used drugs such as triptans at that time[18]. Specific drugs for migraine, such as triptans, are few in number, which makes many people reluctant to see a doctor. Among all acute treatment drugs, the prescription of these specific drugs is less than 3.3%, and it is especially difficult to find them in rural clinics [19]. With our country's more and more understanding of migraine, the age-adjusted incidence and prevalence rate began to rise again between 2000 and 2005. This situation may be due to the popularization of clinical guidelines and the use of triptan therapy, which has brought some slow improvement[20] , although direct supporting evidence remains limited. From 2016 to 2019, we see that the growth rate has become faster again, which is just the time when the "Healthy China 2030" initiative began to be implemented. The plan pays special attention to making more people enjoy medical services and increases the services of specialists. From 2019 to 2021, the data decreased slightly, which is probably due to the influence of COVID-19 epidemic. During that time, the number of people going to the hospital outpatient service decreased obviously. Although the number of people who see online doctors has increased, the accuracy of online diagnosis is not so high, so the data we see has decreased, which may not be that there are fewer migraine patients, but that some cases have not been recorded[21].”

(Revised manuscript, Lines 201–217)

3. Explanation of changes:

1996–2000 decline: Linked to underdiagnosis, misclassification, and very low availability of migraine-specific therapies such as triptans (Lines 206–212).

2000–2005 increase: Attributed to improved diagnostic awareness, dissemination of national guidelines, and the gradual introduction of triptan therapy (Lines 212-215).

2016–2019 acceleration: Contextualized with the Healthy China 2030 initiative, which expanded healthcare access and strengthened neurology services (Lines 215–218).

2019–2021 decline: Explained as an artifact of reduced outpatient visits during the COVID-19 pandemic, combined with limited accuracy of telemedicine diagnosis (Lines 218–223).

4. Purpose and significance:

These revisions address the reviewer’s concern by embedding epidemiological inflection points within China’s historical clinical and policy context. This provides readers with a clearer understanding of how diagnostic practices, treatment availability, and health policy developments may have influenced the observed trends, enhancing both interpretability and public health relevance.

**Reviewer #1 point 6: F.** Superficial Clinical Interpretation

Sex and age-related differences are discussed briefly, but without meaningful reference to pathophysiological mechanisms. Notably:

CGRP biology, trigeminovascular pathways, and cortical spreading depression are not mentioned.

The increase in incidence beginning at age 5–9 is not discussed in the context of pediatric migraine.

Recommendation: Enrich discussion with insights from headache neuroscience and pediatric migraine epidemiology.

Author response:

We thank the reviewer for this valuable suggestion. In the revised manuscript, we have expanded the Discussion section to incorporate detailed pathophysiological explanations (CGRP biology, trigeminovascular pathway, and cortical spreading depression) and to contextualize the observed increase in incidence among children with reference to pediatric migraine epidemiology.

1. Original text (before revision):

“The epidemiological characteristics of migraine exhibit significant gender differences, with the burden of the disease being markedly higher in female patients compared to males. … By 2050, the growth rates of incidence, prevalence, and disability-adjusted life years (DALYs) for male migraine patients are projected to surpass those of females. This phenomenon may reflect the escalating health challenges faced by men in modern society, such as high-intensity physical labor, heavy work-related stress, and relatively weaker awareness of personal health management.”

(Original manuscript, Lines 159–177)

This version only briefly discussed gender differences and social factors, without reference to neurobiological mechanisms or pediatric migraine.

2. Revised text (after revision):

“Trigeminovascular pathway plays a central role in migraine attack. This pathway originated from the sensory neurons in the trigger ganglion, and their peripheral branches connected with intracranial blood vessels and dura mater, while the central part extended to the trigger nucleus caudalis in the brain stem. When these afferent fibers are activated, calcitonin gene-related peptide (CGRP) and other neuropeptides will be released in meningeal blood vessels, leading to vasodilation, plasma protein exudation and mast cell degranulation[23, 24]. Together, these mechanisms inflame our nerves, so the pain signal spreads even more. In this way, the trigeminal nerve becomes particularly sensitive, so that those "pain signals" are constantly transmitted to the brain stem and cerebral cortex, so the headache can not be cured. Sex hormones-especially Estrogen-can also affect this process. Estrogen can affect CGRP signal in many ways, for example, it can promote gene transcription, help peptide release, and increase the number of receptor CLR and RAMP1[25, 26] . It also affects migraine-related neurotransmission by regulating serotonin and glutamate activity[12, 27, 28] . The changes in estrogen levels in our bodies can explain why migraines increase in adolescence, become the most severe in young adults, and then decrease after menopause. Monoclonal antibodies and small molecular antagonists that specifically deal with CGRP- trigeminal neurovascular pathway are really useful in clinic, which tells us that this pathway is super important in the process of migraine[29]. This effect can help us understand why migraine becomes significantly more common after puberty and why many women report that migraine is often related to menstruation. They also make us understand that the therapeutic effect will vary with gender, for example, women may have different reactions to CGRP-targeted interventions. Although females presently bear a higher migraine burden, demographic projections suggest that by 2050, the rates of increase in incidence, prevalence, and Disability-Adjusted Life Years (DALYs) among males will exceed those in females [30]. This may reflect higher work stress and limited health awareness among men [30], cultural barriers to reporting and treatment[31], and insufficient help-seeking [32], underscoring the need to reconsider migraine as a “female disease”[16] and to strengthen male-focused prevention and early intervention [7].

Another pivotal mechanism involved in migraine pathophysiology is cortical spreading depression (CSD), characterized by a slowly advancing wave of neuronal and glial depolarization followed by a sustained suppression of cortical activity. CSD is widely recognized as the electrophysiological basis underlying the migraine aura [33]. By disturbing the ion balance in our body, and letting some exciting signal substances such as glutamate and potassium escape, CSD will activate the pain receptors on the meninges through a system called trigeminovascular, thus establishing a connection between cerebral cortex overexcitation and headache onset[34]. Patients with migraine with aura may not only have special symptoms, such as blurred vision or abnormal sensation before headache, but also face higher long-term cerebrovascular risk. This is because CSD is thought to destroy blood-brain barrier and increase the risk of stroke[35, 36]. This mechanistic framework explains why cortical hyperexcitability is not merely an epiphenomenon but an active contributor to both the phenotype and comorbid risks of migraine.

In addition to adult patterns, our findings revealed that migraine incidence can rise as early as 5–9 years of age. Consistent with this, Meng et al. reported that many cases actually begin in childhood or adolescence[37] , with prevalence reaching 7–10% among school-aged children and up to 20% in adolescents[38]. However, children's migraine is often not found by doctors. Because it is often not the kind of headache that we usually know, but it will turn into a strange appearance such as stomachache, cyclic violence, or sudden dizziness[39]. Recognizing these atypical manifestations is crucial for achieving timely diagnosis and reducing the burden associated with inadequately treated childhood migraine. Headache disorders that emerge during adolescence may persist or even worsen over time, potentially developing into chronic and refractory forms that impose substantial burdens on both individuals and society[29]. Environmental triggers such as academic stress, sleep deprivation, and excessive electronic device use further exacerbate the risk of migraine onset in children and adolescents[40], with excessive device use identified as a significant factor[41]. Migraine is the leading cause of health loss between ages 15 and 49[42], and onset typically occurs before 18, challenging the prior view of adult onset [43]. The age range of 15–39 is critical for education, career, and social development, with heightened susceptibility[44], while individuals aged 35–44 face dual career and family pressures contributing to peak prevalence and YLDs [7]. As global aging accelerates, children and adolescents will remain a key demographic, requiring targeted prevention and treatment to reduce social and economic burdens [37]. Young and middle-aged patients are also disproportionately affected by stigmatization, necessitating improved awareness, patient dignity, and policy interventions [45].”

(Revised manuscript, Lines 231-288)

3. Explanation of changes:

CGRP and trigeminovascular pathway: Added detailed description of neurogenic inflammation, the role of estrogen in CGRP modulation, and the therapeutic relevance of CGRP-targeted drugs (Lines 234–243).

Cortical spreading depression (CSD): Introduced as the electrophysiological basis of migraine aura and its link to headache initiation and cerebrovascular risk (Lines 257–266).

Pediatric migraine: Discussed the early onset of incidence (age 5–9), underdiagnosis, atypical presentations, and importance of timely recognition (Lines269–276).

4. Purpose and significance:

These revisions strengthen the clinical interpretation by linking epidemiological findings to underlying biological mechanisms and pediatric migraine epidemiology. This not only enriches the discussion but also enhances the translational relevance of the study, providing readers with a more comprehensive understanding of sex- and age-related differences in migraine burden.

**Reviewer #1 point 7: G.** Speculative Interpretations of Lifestyle Factors

Attributing trends to screen time, stress, and diet is speculative and not empirically tested.

Recommendation: Rephrase such points as hypotheses or broader societal factors, not study findings.

Author response:

We appreciate the reviewer’s thoughtful comment. We have revised the Discussion to reframe our interpretation of lifestyle factors such as screen time, stress, and diet. These points are now presented as potential hypotheses or broader societal influences, rather than definitive study findings.

1. Original text (before revision):

“Modifications in lifestyle and occupational factors, such as prolonged sedentary behavior, increased stress, the widespread consumption of high-fat and high-protein diets, and elevated use of electronic devices, have been identified as triggers that can intensify the severity of headaches.”

(Original manuscript, Lines 58–60)

This version suggested a direct causal link between lifestyle factors and observed trends, which was speculative.

2. Revised text (after revision):

“Wider social and lifestyle changes-including more and more sedentary jobs, rising stress levels, changing eating habits to Western diet featuring high-sugar foods and processed foods, and increasing dependence on electronic devices-have been identified as potential triggers of migraine risk in previous studies. These factors may provide a meaningful background for us to understand the increasing global migraine burden, and point out the important direction of future empirical research[8].”

(Revised manuscript, Lines 57–62)

3. Explanation of changes:

Rephrasing: Instead of stating these factors as triggers, we now cite them as “in previous studies” (Lines 60).

Hypothesis framing: We explicitly noted that these are “potential triggers” and should be regarded as “meaningful background” rather than confirmed findings (Lines 60-61).

Future direction: We added that such factors represent “important direction of future empirical research,” shifting the emphasis from conclusion to hypothesis ( Lines 62).

4. Purpose and significance:

These revisions remove overstatement and ensure that lifestyle factors are presented cautiously, in line with the available evidence. This aligns with the reviewer’s recommendation and strengthens the scientific rigor of the discussion by distinguishing between study findings and broader hypotheses.

**Reviewer #1 point 8:** H. Missing Limitations Section

Most critically, the manuscript lacks any discussion of limitations, which is required by PLOS ONE.

Recommendation: Include a paragraph acknowledging:

Use of GBD-modeled data rather than clinical registries,

Potential for misclassification or underdiagnosis (especially in rural or male populations),

No geographic stratification within China,

Unmeasured confounders (e.g., urbanization, air pollution),

Constraints inherent in APC modeling.

Author response:

We thank the reviewer for this important observation. In the revised manuscript, we have added a dedicated Limitations section to explicitly acknowledge the potential weaknesses of our study.

1. Original text (before revision):

The original manuscript did not contain a Limitations section.

2. Revised text (after revision):

“Several limitations should be acknowledged in this study. First, all estimates were generated from the Global Burden of Disease (GBD) database, which, although incorporating Chinese data sources—surveys, registries, hospital records, and insurance claims—relies on statistical modeling rather than direct data analysis. As a result, the outcomes may not precisely represent primary national data, and biases arising from input quality or model specification could yield systematic error. Therefore, these results may not fully represent the real situation of our country, and if the quality of input data is not good or the model setting is problematic, systematic errors may occur. Second, some cases may not be correctly recorded or classified by doctors, especially in rural areas, and among men who are not convenient to see a doctor. Third, our analysis did not take into account the huge differences between different places, cities and rural areas, and between the rich and the poor in China. Because we did not study these differences carefully, the results may be biased. Fourthly, some important factors, such as the change of people's living habits, air pollution and urbanization, are not included in the analysis. Finally, the age–period–cohort model, while useful for exploring temporal dynamics, relies on assumptions that constrain causal interpretation. So when we look at our research results, we should remember these imperfections, which also shows that we need to test and expand our findings with more detailed clinical data from more people in the future.”

(Revised manuscript, Lines 205–320)

3. Explanation of changes:

GBD-modeled data: Added a statement that reliance on GBD estimates, rather than direct registries, may bias results (Lines 305–311).

Misclassification/underdiagnosis: Noted that rural and male populations may be particularly affected (Lines 312–313).

Geographic stratification: Acknowledged lack of provincial- or urban–rural-level analysis (Lines 313–315).

Unmeasured confounders: Included examples such as urbanization, air pollution, and lifestyle factors (Lines 316–317).

APC model constraints: Highlighted that APC modeling relies on assumptions limiting causal interpretation (Lines 317–318).

4. Purpose and significance:

By adding a dedicated Limitations section, the manuscript now conforms to PLOS ONE requirements and provides readers with a transparent appraisal of potential weaknesses. This strengthens the credibility of the study, helps contextualize the findings, and indicates directions for future research.

**Reviewer #1 point 9: Minor Comments**

Language: Consider professional editing for grammar and sentence flow.

Figures: Simplify and ensure that legends clarify key trends.

Data sharing and ethical statements are appropriate.

Author response:

We thank the reviewer for these helpful minor comments. We have addressed them as follows:

1. Original text (before revision):

The original manuscript contained occasional grammatical issues, longer sentences affecting readability, and figure legends that were relatively brief.

2. Revised text (after revision):

Language: The entire manuscript was carefully revised by all co-authors, and grammar and sentence flow were professionally edited to improve clarity and readability. For example, the Introduction and Discussion sections have been rephrased to ensure smoother sentence flow.

Figures: We improved the clarity of the figures by adjusting formatting (e.g., resolution and label readability). In addition, we expanded the legends to clarify key epidemiological trends. For example, the legends to Figure 1 (age-specific prevalence, incidence, and YLDs) and Figure 3 (APC effects) now explicitly state the most affected age groups and highlight sex differences (Supporting Figures S1–S3).

Data sharing and ethics: No changes were needed, as the Data Availability and Ethics statements were already compliant with PLOS ONE requirements (Page 3, Lines 96–100).

3. Explanation of changes:

Language: Improved overall readability and grammar through careful revision and professional editing.

Figures: Enhanced visual clarity by formatting adjustments, and expanded legends to better explain key patterns and sex/age differences.

Data sharing/ethics: Confirmed appropriateness; no revisions required.

4. Purpose and significance:

These adjustments ensure the manuscript meets high standards of clarity, readability, and presentation quality, making it more accessible to a broad readership while maintaining scientific rigor.

**Reviewer #2 point 1:**

Comment:

Among migraine comorbidities, the authors should also mention metabolic conditions such as insulin resistance and obesity (Del Moro L, Rota E, Pirovano E, Rainero I. Migraine, Brain Glucose Metabolism and the "Neuroenergetic" Hypothesis: A Scoping Review. J Pain. 2022 Aug;23(8):1294–1317), as well as cardiovascular diseases (Guidetti D, Rota E, Morelli N, Immovilli P. Migraine and stroke: "vascular" comorbidity. Front Neurol. 2014 Oct 8;5:193; Bigal ME et al. Migraine and cardiovascular disease: a population-based study. Neurology. 2010 Feb 23;74(8):628–35).

Author response:

We thank the reviewer for this excellent suggestion. In the revised manuscript, we have expanded the Introduction to include metabolic and cardiovascular comorbidities of migraine, supported by the suggested references.

1. Original text (before revision):

“In addition to the core symptoms, migraines are frequently associated with comorbid conditions, including anxiety, depression, and hypertension, which exacerbate the overall burden of the disorder.”

(Original manuscript, Lines 52–54)

2. Revised text (after revision):

“Migraine often appears with psychological problems such as anxiety and depression, and it is also related to other physical problems, such as poor metabolism-especially insulin resistance and obesity[2] -and it is also closely related to cardiovascular diseases such as hypertension and stroke[3, 4].”

(Revised manuscript, Lines 50–53)

The corresponding new references are listed in the revised manuscript as:

Del Moro et al., 2022 (Reference 2, Line 341),

Guidetti et al., 2014 (Reference 3, Line 344),

Bigal et al., 2010 (Reference 4,Line 347).

3. Explanation of changes:

Metabolic conditions added: We incorporated insulin resistance and obesity as migraine comorbidities (Lines 52-53; Ref. 2).

Cardiovascular diseases added: We added stroke and cardiovascular disease (Lines 53–54; Ref. 3, Ref. 4,).

Reframed context: We emphasized that migraine is not only a neurological disorder but also a systemic condition with broad clinical and public health implications (Lines 53–55).

4. Purpose and significance:

These additions provide a more comprehensive overview of migraine comorbidities and highlight its systemic nature. By citing Del Moro et al. (2022), Guidetti et al. (2014), and Bigal et al. (2010), the Introduction now better reflects the broader clinical relevance of migraine.

**Reviewer #2 point 2:**

Comment:

I recommend replacing the phrase “high-fat and high-protein diets” with the more general term “Western diet,” as some studies have shown that ketogenic diets, which are high in fat, may actually reduce migraine frequency and severity. Conversely, growing evidence suggests that high-glycemic index foods are the main dietary contributors to increased migraine burden (Del Moro L, Rota E, Pirovano E, Rainero I. Migraine, Brain Glucose Metabolism and the "Neuroenergetic" Hypothesis: A Scoping Review. J Pain. 2022 Aug;23(8):1294–1317).

Author response:

We thank the reviewer for this helpful suggestion. We have revised the Introduction to replace the phrase “high-fat and high-protein diets” with the more appropriate term “Western dietary patterns,” and we emphasized that high-glycemic index foods and processed products are increasingly recognized as contributors to migraine burden. The suggested reference (Del Moro et al., 2022) has been cited.

1. Original text (before revision):

“Modifications in lifestyle and occupational factors, such as prolonged sedentary behavior, increased stress, the widespread consumption of high-fat and high-protein diets, and elevated use of electronic devices, have been identified as triggers that can intensify the severity of headaches.”

(Original manuscript, Lines 57–60)

2. Revised text (after revision):

“Wider social and lifestyle changes-including more and more sedentary jobs, rising stress levels, changing eating habits to Western diet featuring high-sugar foods and processed foods, and increasing dependence on electronic devices-have been identified as potential triggers of migraine risk in previous studies.”

(Revised manuscript, Lines 57-60)

The corresponding new reference is:

Del Moro et al., 2022 (Line 341, Reference 2).

3. Explanation of changes:

Terminology corrected: Replaced “high-fat and high-protein diets” with “Western dietary patterns” (Line 59).

Scientific accuracy improved: Clarified that high-glycemic index foods and processed products are likely dietary contributors (Lines 60).

Citation added: Cited Del Moro et al. (2022) to support this refinement (Line 341, Reference 2,).

4. Purpose and significance:

This revision ensures greater accuracy and avoids misleading implications about fat intake, recognizing that certain high-fat diets (e.g., ketogenic) may have protective effects. By citing Del Moro et al. (2022), we anchor our statement in recent scientific evidence and present a more balanced view of diet-related migraine risk.

**Reviewer #2 point 3:**

Comment:

Although the study provides insightful decomposition analyses illustrating the contribution of demographic and epidemiological factors to the migraine burden, the methodology is not explicitly described. No formal models, equations, or supplementary materials are provided to allow for replication or critical evaluation of the decomposition approach.

Author response:

We thank the reviewer for pointing out this omission. In the revised manuscript, we have expanded the Methods section to provide a more explicit description of the decomposition analysis, including the formal equation, definitions of each component, and a reference to supplementary materials for replication.

1. Original text (before revision):

“Disaggregation analyses were used to calculate the contribution of population growth, ageing, and epidemiological changes to the burden of disease.”

(Original manuscript, Lines 33–35)

“R software version 4.4.1 was employed for data visualization, decomposition analysis, and age-period-cohort (APC) analysis. Joinpoint software was used to compute the annual percent change (APC), average annual percent change (AAPC), and 95% confidence intervals (CI) for the migraine disease burden.”

(Original manuscript, Lines 77–80)

This version did not describe the methodology in sufficient detail and lacked equations.

2. Revised text (after revision):

“We used a powerful analysis method in R software (version 4.4.1) to calculate how population growth, aging people and changes in disease patterns affected migraine burden from 1990 to 2021. As the previous study [14] said, this method can separate the influence of population structure from the influence of different ages and gender incidence changes. The total change in cases or rates (ΔY) was expressed as the aggregation of three components:

ΔY = M_p_ + M_a_ + M_m_,

where M_p_ represents the contribution of population growth, M_a_ reflects the contribution of population ageing (i.e., changes in age structure), and M_m_ captures epidemiological change, defined as shifts in age- and sex-specific incidence, prevalence, or YLDs rates after controlling for population size and age structure. The detailed mathematical formulation of the decomposition model, including the definition of interaction terms and their allocation, is provided in **Supplementary Text S1**.”

(Revised manuscript, Lines 104–115)

3. Explanation of changes:

Formalization: Added the explicit equation ΔY = M_p_ + M_a_ + M_m_ (Lines 109).

Supplementary material: Pointed readers to Supplementary Text S1 for the full mathematical formulation (Lines 115).

4. Purpose and significance:

These revisions make the decomposition methodology transparent and replicable, addressing the reviewer’s concern. By providing a formal equation, clear definitions, and supplementary material, we allow readers to critically evaluate and reproduce the analysis, thereby strengthening the methodological rigor of the manuscript.

**Reviewer #2 point 4:**

Comment:

There is a discrepancy between the figures reported in the text and those in Table 1. For example, the text states “the total number of migraine cases in China was 18,475,280,” while Table 1 reports “184,752,280”.

Author response:

We thank the reviewer for carefully noting this error. We have corrected the figures in the Results section to match those presented in Table 1.

1. Original text (before revision):

“As shown in Table 1, in 2021, the total number of migraine cases in China was 18,475,280 …”

(Original manuscript, Lines 85–86)

This incorrectly reported the number of cases, dropping a digit compared with Table 1.

2. Revised text (after revision):

“In 2021, the total number of migraine cases in China reached 184,752,280, comprising 70,036,223 males and 114,716,057 females.”

(Revised manuscript, Lines 130)

This correction ensures consistency with Table 1.

3. Explanation of changes:

Numerical correction: Corrected “18,475,280” → “184,752,280” (Line 130).

Clarity: We also added the breakdown by sex (70,036,223 males and 114,716,057 females), consistent with Table 1 (Lines 142).

4. Purpose and significance:

This revision ensures consistency between the text and Table 1. Accurate reporting of case numbers is essential for clarity, reproducibility, and credibility of the study.

**Reviewer #2 point 5:**

Comment:

Table 1 should be corrected: values should be reported with only one decimal place.

Author response:

We thank the reviewer for this helpful suggestion. In the revised manuscript, we reformatted Table 1 so that all values are now reported with only one decimal place for consistency and clarity.

1. Original text (before revision):

Table 1 Migraine cases and age-standardized prevalence, incidence, and YLDs rate in all age groups in China in 2021

Values in the original Table 1 were reported with multiple decimal places (e.g., “11777.51,” “975.61,” “443.65”).

(Original manuscript, Lines 88–91)

2. Revised text (after revision):

Table 1 Migraine cases and age-standardized prevalence, incidence, and YLDs rates in all age groups in China in 2021

Values in the revised Table 1 are now reported with one decimal place (e.g., “11,777.5,” “975.6,” “443.7”).

(Revised manuscript, Lines 142; Table 1)

3. Explanation of changes:

Decimal adjustment: Reduced numerical precision to one decimal place across all values in Table 1.

Consistency: Ensured all prevalence, incidence, and YLDs rates are consistently formatted.

Clarity: Retained significant figures sufficient for epidemiological interpretation while improving readability.

4. Purpose and significance:

This revision improves the presentation quality of Table 1, making the data clearer and more reader-friendly while retaining appropriate precision for scientific reporting.

**Reviewer #2 point 6:**

Comment:

In the main text, epidemiological data should be rounded and reported without decimal places to improve clarity.

Author response:

We sincerely thank the reviewer for this thoughtful suggestion. We carefully considered the presentation of numerical data in the main text, as clarity and consistency are indeed crucial. In the revised manuscript, we have reduced the number of decimal places from two to one for all age-standardized rates, in order to improve readability. We chose to retain one decimal place (rather than rounding to integers) to maintain consistency with Table 1 and to ensure that readers can directly cross-reference values between the text and the table without confusion. This approach also reflects common practice in epidemiological reporting, where one decimal is often used to convey the precision of modeled estimates without overstating accuracy.

1. Original text (before revision):

“The age-standardized prevalence rate of migraine was 11,777.51 per 100,000 population, with 8,781.96 in males and 14,959.04 in females. The incidence rate was 975.61 per 100,000 population, with 722.18 in males and 1,252.68 in females. The YLDs rate was 443.65 per 100,000 population, with 341.31 in males and 552.65 in females.”

(Original manuscript, Results – Descriptive Analysis, Lines 85-97)

2. Revised text (after revision):

“The age-standardized prevalence rate of migraine was 11,777.5 per 100,000 population, with 8,782.0 in males and 14,959.0 in females. The incidence rate was 975.6 per 100,000 population, with 722.2 in males and 1,252.7 in females. The YLDs rate was 443.7 per 100,000 population, with 341.3 in males and 552.7 in females.”

(Revised manuscript, Results – Descriptive Analysis, Lines 134–136)

3. Explanation of changes:

Decimals reduced but retained: Changed from two decimal places to one (e.g., 11,777.51 to 11,777.5), thereby improving readability while still conveying adequate precision.

Consistency maintained: Applied one decimal place consistently in both the main text and Table 1, ensuring that values match across sections.

Scientific accuracy preserved: One decimal place is a balanced approach—providing sufficient epidemiological precision without implying false accuracy.

4. Purpose and significance:

These adjustments enhance the clarity of numerical presentation while maintaining internal consistency and methodological rigor. We believe this approach represents a reasonable compromise between readability and scientific accuracy. **If the editor prefers, we are also willing to round all values to integers in the main text.**

**Reviewer #2 point 7:**

Comment:

The concept of “epidemiological change” is vague and not adequately defined. The authors do not explain which variables were used to represent these changes or how they were measured.

Author response:

We sincerely thank the reviewer for this helpful comment. We agree that the original text did not sufficiently define the concept of “epidemiological change.” In the revised manuscript, we have clarified its meaning in the Methods section and specified how it was operationalized in our decomposition analysis.

1. Original text (before revision):

“Epidemiological changes have contributed to the variations in migraine burden in China;”

(Original manuscript, Lines 128–129)

“Additionally, epidemiological changes have played a significant role in driving the increase in migraine YLDs, with a particularly pronounced impact in females.”

(Original manuscript, Lines 136–137)

This version simply mentioned “epidemiological changes” without definition.

2. Revised text (after revision):

“Epidemiological change—defined as variation in age- and sex-specific incidence, prevalence, or YLDs after accounting for changes in population size and structure—also contributed to the observed increases.”

(Revised manuscript, Methods – Decomposition Analysis, Lines 174–176)

3. Explanation of changes:

Definition added: We explicitly defined “epidemiological change” as shifts in age- and sex-specific incidence, prevalence, or YLDs rates that remain after controlling for population size and demographic structure (Lines 174–176).

Operationalization clarified: Explained that epidemiological change was measured by the residual component of the decomposition equation (ΔY = M_p_ + M_a_ + M_m_), where M_m_ represents the effect of epidemiological change (Lines 111).

Contextualization: In the Results section, we highlighted how epidemiological change influenced migraine trends, particularly among females (Lines 176–180).

4. Purpose and significance:

These revisions resolve the reviewer’s concern by providing a clear definition and methodological basis for “epidemiological change.” This improves the transparency and reproducibility of the decomposition analysis, and ensures that readers can better understand how demographic versus epidemiological factors contributed to the changing burden of migraine in China.

**Reviewer #2 point 8:**

Comment:

The Limitations section should explicitly mention that the study relies entirely on modeled estimates from the Global Burden of Disease, without comparison to real-world national data (e.g., Chinese surveillance systems, hospital admissions, prescription databases). GBD estimates are derived from complex models and may overestimate or underestimate true values, especially in countries with limited high-quality local data. This could introduce uncontrolled systematic bias.

Author response:

We sincerely thank the reviewer for this important observation. We agree that it is critical to acknowledge the reliance on modeled GBD estimates and the potential for systematic bias. Accordingly, we have revised the Limitations section to explicitly state this limitation.

1. Original text (before revision):

The original manuscript did not contain a dedicated Limitations section.

2. Revised text (after revision):

“This study has several limitations. First, it relied entirely on modeled estimates from the Global Burden of Disease (GBD) database. Although GBD incorporates Chinese data sources such as surveys, registries, hospital records, and insurance claims, these are synthesized through statistical models rather than analyzed directly. Thus, results may not fully reflect raw national data, and biases in input quality or modeling assumptions could introduce systematic error.”

(Revised manuscript, Limitations, Lines 305–311)

3. Explanation of changes:

Explicit acknowledgment: Added that the study relied exclusively on modeled GBD estimates (Line 305-306).

Lack of direct comparison: Clarified that no direct comparison with real-world Chinese surveillance, hospital, or prescription data was possible (Lines 310–311).

Potential bias: Stated that GBD’s reliance on modeling may introduce uncontrolled systematic bias, especially in countries with limited high-quality local data (Lines 308–310).

4. Purpose and significance:

This revision directly addresses the reviewer’s concern by transparently acknowledging the potential limitations of using modeled GBD estimates. By highlighting the lack of real-world data comparison and the possibility of systematic bias, the manuscript provides readers with a more balanced understanding of the strengths and limitations of the study, thereby enhancing credibility and methodological rigor.

**Reviewer #2 point 9:**

Comment:

China is geographically and socioeconomically heterogeneous, but the study does not examine regional differences (e.g., by province), urban vs. rural settings, or socioeconomic strata. This is another potential source of bias that should be acknowledged.

Author response:

We sincerely thank the reviewer for this constructive comment. We agree that failing to account for China’s regional and socioeconomic heterogeneity is a limitation. We have revised the Limitations section to explicitly acknowledge this issue.

1. Original text (before revision):

The original manuscript did not include discussion of China’s geographic or socioeconomic diversity as a limitation.

2. Revised text (after revision):

“Third, our analysis did not take into account the huge differences between different places, cities and rural areas, and between the rich and the poor in China. Because we did not study these differences carefully, the results may be biased.”

(Revised manuscript, Limitations, Lines 313–315)

3. Explanation of changes:

Geographic heterogeneity: Added acknowledgment that the study did not analyze regional/provincial differences within China.

Urban–rural divide: Explicitly stated that we did not account for urban vs. rural populations.

Socioeconomic strata: Noted the absence of stratification by socioeconomic status.

Potential bias: Emphasized that the lack of stratified analysis may introduce additional bias.

4. Purpose and significance:

This revision strengthens the transparency of the Limitations section by highlighting the omission of regional and socioeconomic stratification. Acknowledging this limitation provides readers with a more balanced understanding of the study’s scope and encourages future research to address these important disparities.

**Reviewer #2 point 10:**

Comment:

The discussion section includes several topics that are only tangentially related to the study’s results, such as calcitonin gene-related peptide (CGRP) therapy, estrogen’s contribution, and the framing of migraine as a "female disease". These should be either removed or significantly reduced, as they are not directly supported by the findings of this study.

Author response:

We sincerely thank the reviewer for this thoughtful comment. We fully understand the concern that extensive discussion of CGRP therapy, estrogen, and the social framing of migraine may appear tangential to the direct results of our study. At the same time, another reviewer suggested expanding the discussion of sex- and age-related differences with reference to neurobiological mechanisms, including estrogen and CGRP biology.

To balance these perspectives, we have substantially shortened and streamlined these sections. In the revised manuscript, we now only briefly mention estrogen and CGRP as possible biological explanations for the sex-related differences observed in our data, while removing the detailed mechanistic descriptions and therapeutic discussions. The framing of migraine as a “female disease” has also been condensed to a single sentence highlighting the risk of under-recognition in men.

1. Original text (before revision):

Contained long discussions of estrogen mechanisms, CGRP-targeted therapy, and the “female disease” narrative.

(Original manuscript, Discussion, Lines 159–177)

2. Revised text (after revision):

We also found a big difference between men and women. The burden of illness of women is much heavier than that of men [22]. These sex- and age-related variations in migraine burden can be explained by well-established neurobiological mechanisms[23]. (Lines 227–230)

This may reflect higher work stress and limited health awareness among men [30], cultural barriers to reporting and treatment[31], and insufficient help-seeking [32], underscoring the need to reconsider migraine as a “female disease”[16] and to strengthen male-focused prevention and early intervention [7]. (Revised manuscript, Discussion, Lines 253-256)

3. Explanation of changes:

CGRP therapy: Reduced to a brief contextual mention, without detailed discussion.

Estrogen: Condensed into one sentence as a potential explanation for sex differences.

“Female disease” framing: Reduced to a brief acknowledgment of under-recognition in men.

4. Purpose and significance:

These revisions ensure that the Discussion remains closely tied to our findings while acknowledging key biological mechanisms in a concise and balanced way. We believe this approach appropriately addresses the concerns of both reviewers and strengthens the overall focus of the manuscript.

We would like to sincerely thank the reviewers and the editor once again for their thorough and constructive comments, which have greatly improved the quality and clarity of our manuscript. We have carefully addressed each point raised, revised the manuscript accordingly, and highlighted all changes in the revised version. We believe that the manuscript has been substantially strengthened in terms of scientific rigor, methodological transparency, and overall readability.

We remain very grateful for the opportunity to revise and resubmit our work, and we respectfully submit this revised version for your further consideration. We hope that the revisions meet with your approval, and we are of course willing to make any additional changes that the editor or reviewers may recommend.

Sincerely,

Yuting, Huang

On behalf of all authors
