# Supplementary material for: Trends and age-period-cohort analysis of migraine incidence in China from 1990 to 2021
Source: PLoS One. 2026 Feb 13;21(2):e0338930. doi: 10.1371/journal.pone.0338930 (PMC12904424; doi:10.1371/journal.pone.0338930)
Supplement: Attachment — Submitted filename: Response to Reviewers.docx [file pone.0338930.s005.docx]

Dear Editors and Reviewers,

We thank the reviewers for their constructive and insightful comments. Below, we provide a detailed, line-by-line response. All revisions have been incorporated into the revised manuscript, and line numbers refer to the updated version.

Reviewer #1 — Response

Point 4. Methodological Transparency in APC Modeling

Reviewer comment:

“The author has described the Holford method and the Biowinford platform used for APC modeling. However, the lack of diagnostics (e.g., AIC, BIC) is acknowledged but not fully explained.

Suggested Improvement: The author should further explain the impact of missing diagnostics on the robustness of the model and clarify how the Holford method is widely validated despite the lack of diagnostics.

Conclusion: Mostly addressed, but further clarification required.”

Author Response:

We thank the reviewer for emphasizing this important methodological issue. Following the suggestion, we have substantially expanded the explanation of why AIC/BIC diagnostics are not applicable in the Holford APC framework and added literature-supported justification of the robustness and validation of this approach.

Summary of Revision

Revised section: Methods “Age–Period–Cohort (APC) Analysis”

Revised manuscript lines: Line 117–121,124-126.

Original text (for reviewer reference):

Lines 117–127 of original submission:

“Age–Period–Cohort (APC) analysis was performed using the Biowinford online platform

(http://biowinford.site:3838/trial/). The platform’s identification strategy employs the constraint based Holford method, which resolves the inherent linear dependency among age, period, and cohort effects by imposing parameter constraints on period and cohort terms. This approach aligns with the methodology implemented in the NIH’s APC web tool (https://analysistools.cancer.gov/apc/), though the NIH version is broader in scope and not tailored to migraine or GBD-specific data. The Biowinford output excludes certain model diagnostics, including residual analysis, Akaike Information Criterion (AIC), Bayesian Information Criterion (BIC), and Variance Inflation Factor (VIF). Although this represents a methodological limitation, the consistency of the identification strategy, case definitions, and data sources with established APC frameworks supports the robustness and interpretive value of the resulting trend estimates.”

Revised text (new version with literature insertion):

Line 117–121 (revised manuscript):

“Although the platform does not provide conventional diagnostics such as AIC, BIC, or residual analysis, this does not compromise methodological robustness, because likelihood-based information criteria are not applicable under APC’s exact linear dependency structure—an established property documented in foundational APC methodological work[15].

Line 124–126 (revised manuscript):

“Empirical applications consistently demonstrate stable and reproducible age, period, and cohort trend estimates across large population datasets, supporting the reliability of APC results even in the absence of traditional diagnostics[16, 17].”

Inserted references:

Line 388–396 (revised manuscript):

15.	Rosenberg PS, Anderson WF. Age-period-cohort models in cancer surveillance research: ready for prime time? Cancer Epidemiol Biomarkers Prev. 2011;20(7):1263-8. 2011/05/26. https://doi.org/10.1158/1055-9965.Epi-11-0421. PMID: 21610223.

16.	Rosenberg PS, Check DP, Anderson WF. A web tool for age-period-cohort analysis of cancer incidence and mortality rates. Cancer Epidemiol Biomarkers Prev. 2014;23(11):2296-302. 2014/08/26. https://doi.org/10.1158/1055-9965.Epi-14-0300. PMID: 25146089.

17.	Luo L, Hodges JS. Constraints in Random Effects Age-Period-Cohort Models. Sociol Methodol. 2020;50(1):276-317. 2020/08/01. https://doi.org/10.1177/0081175020903348. PMID: 36644415.

In response to the reviewer’s comment on APC model diagnostics, we have revised the Methods section to clarify that the Biowinford platform implements the constraint-based Holford estimable-function framework, in which likelihood-based selection criteria such as AIC and BIC are not applicable under the exact linear dependency among age, period, and cohort[15]. We further note that this approach is identical to that of the NIH APC web tool and has been extensively validated in epidemiologic and cancer surveillance studies, consistently yielding stable and reproducible temporal trend estimates[16,17]. These additions explain why the absence of AIC/BIC output does not compromise the robustness of our APC estimates.

Reviewer #2 — Response

Comment: “The authors have adequately addressed all the comments.”

Response: We thank Reviewer #2 for the positive evaluation and are grateful for the support.

We would like to express our sincere gratitude to the reviewers for their thoughtful, constructive, and highly valuable comments. Your careful evaluation and insightful suggestions have substantially enhanced the rigor, clarity, and overall quality of our manuscript. We have revised the manuscript accordingly and addressed each point in detail. We remain deeply appreciative of the reviewers’ time and expertise, and we would be pleased to provide any further clarification or additional modifications if required.

Sincerely,

Yuting, Huang

on behalf of all authors
